# Prolonged SARS-CoV-2 Infection in Patients Receiving Anti-CD20 Monoclonal Antibodies: A Diagnostic Challenged by Negative Nasopharyngeal RT-PCR and Successful Treatment with COVID-19 High-Titer Convalescent Plasma

**DOI:** 10.3390/v15112220

**Published:** 2023-11-07

**Authors:** Léa Da Silva, Timothée Klopfenstein, Vincent Gendrin, Julien Clouet, Lynda Toko, Quentin Richier, Thomas Leriche, Raoul Nicolas, Alexis Queijo, Nour Sreiri, Karine Lacombe, Souheil Zayet

**Affiliations:** 1 Infectious Diseases Department, Nord Franche-Comté Hospital, 90400 Trevenans, France; dasilvalea@hotmail.fr (L.D.S.); vincent.gendrin@hnfc.fr (V.G.); lynda.toko@hnfc.fr (L.T.); alexis.queijo@edu.univ-fcomte.fr (A.Q.); sreiri5nh@gmail.com (N.S.); 2 Infectious Diseases Department, Assistance Publique Hopitaux de Paris, Saint-Antoine Hospital, 75012 Paris, France; quentin.richier@aphp.fr (Q.R.);; 3 Rheumatology Department, Nord Franche-Comte Hospital, 90400 Trevenans, France; thomas.leriche@hnfc.fr; 4 Pneumologie Department, Nord Franche-Comte Hospital, 90400 Trevenans, France; raoul.nicolas@hnfc.fr; 5 INSERM, Pierre Louis Institute of Epidemiology and Public Health (IPLESP), Sorbonne University, 75646 Paris, France

**Keywords:** convalescent plasma, COVID-19, humoral immunity, ocrelizumab, rituximab, SARS-CoV-2

## Abstract

We highlighted in this current paper similar prolonged respiratory presentation with COVID-19 pneumonia in four severely immunocompromised patients currently being treated with anti-CD20 monoclonal antibodies (mAbs), such as ocrelizumab and rituximab, for multiple sclerosis or rheumatoid polyarthritis. Real-time reverse transcription-polymerase chain reaction on a nasopharyngeal swab specimen was negative in all patients. SARS-CoV-2 infection was confirmed from bronchoalveolar lavage fluid. A high titer of post-vaccine COVID-19 convalescent plasma was administered with complete recovery in all patients.

## 1. Key Bullet Points

Immunocompromised patients with B-cell depletion agents are at risk for persistent SARS-CoV-2 infection.

Negative tests for SARS-CoV-2 pneumonia based on RT-PCR cannot rule out this diagnosis.

We reported a case series of prolonged SARS-CoV-2 infection (with negative nasopharyngeal RT-PCR result) in patients receiving anti-CD20 mAbs who recovered after administration of high-titer post-vaccine COVID-19 convalescent plasma.

COVID-19 convalescent plasma is a promising approach through the transfer of neutralizing antibodies specific to SARS-CoV-2 in patients with B-cell immunosuppression and persistent viral shedding.

## 2. Introduction

Immunocompromised patients treated with B-cell depletion treatment (e.g. anti-CD20 monoclonal antibodies (mAbs)) have an increased susceptibility to develop severe acute respiratory syndrome coronavirus 2 (SARS-CoV-2) persistent viral shedding [1]. SARS-CoV-2 infection with respiratory presentation cannot be excluded after performing nasopharyngeal SARS-CoV-2 real-time reverse transcription-polymerase chain reaction (RT-PCR) [2]. Treatments for prolonged coronavirus disease 2019 (COVID-19) are rarely described and there are a lack of recommendations [3,4,5].

In this particular population of patients undergoing anti-CD20 therapy, the T-cell response is conserved [6,7,8], present also against variants [9], and detectable by commercial tests [10]. ‘Prolonged’ SARS-CoV-2 infection was defined as confirmed COVID-19 with symptoms persisting for 1 month or more. We reported herein four cases of confirmed prolonged SARS-CoV-2 infection (despite negative nasopharyngeal RT-PCR result) in patients receiving anti-CD20 mAbs who recovered after administration of high-titer post-vaccine COVID-19 convalescent plasma (CCP).

## 3. Results 

**Figure 1:**
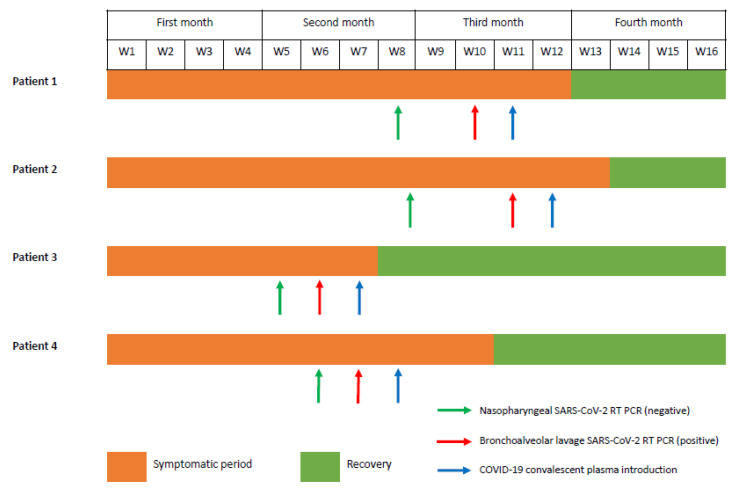
Timeline in patients with prolonged SARS-CoV-2 infection with recovery after COVID-19 convalescent plasma transfusion.

Three of four patients were females with a median age of 54 (43–65) years. All patients were receiving anti-CD20 mAbs, such as ocrelizumab and rituximab, for multiple sclerosis (*n* = 3) and rheumatoid polyarthritis (*n* = 1), respectively. Symptoms onset began 48 (33–58) days before hospitalization with fever and upper respiratory symptoms (Figure 1 and Table 1).

**Figure 2:**
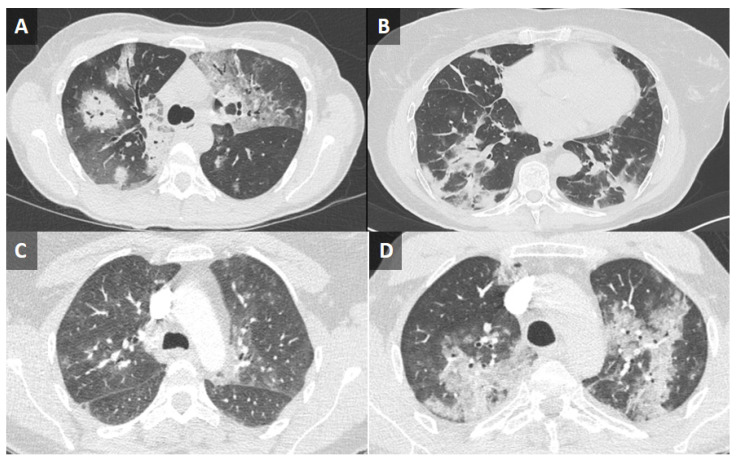
Thoracic computed tomography scan in patients with B-cell depletion and prolonged SARS-CoV-2 infection. (**A**) Ground-glass interstitial syndrome with a crazy paving, predominant on the left upper lobe, and alveolar condensation in the right lower lobe with pleural effusion (Patient 1). (**B**) Bilateral ground-glass opacities with sequelae lesions (Patient 2). (**C**) Subpleural ground-glass opacities with a basal distribution (Patient 3). (**D**) Multiple bilateral foci of ground-glass opacities, affecting all lobes except the right upper lobe (Patient 4).

Real-time reverse transcription-polymerase chain reaction (RT-PCR) on nasopharyngeal swab specimen (TaqPath™ COVID-19 CE-IVD RT-PCR Kit, ThermoFisher Scientific, Waltham, MA, USA) and SARS-CoV-2 serologies (chimiluminescence, Liaison^®^ XL) for IgG (anti-RBD, anti-NTD, anti-Interface) were negative in all patients. Pulmonary computed tomography (CT) scans showed bilateral ground-glass opacities in all patients (Figure 2). SARS-CoV-2 infection was confirmed from bronchoalveolar lavage (BAL) fluid by performing DiagCORE (Maryland, USA) ^®^ Respiratory Panel 2—SAT Dx. CCP was administered in all patients (a total of four units) with a complete recovery without relapse after a mean of 73 days of follow-up.

### 3.1. Case 1

A 43-year-old female with a past history of rheumatoid arthritis treated with rituximab presented with daily fever for two months, chills, and respiratory symptoms such as dypnea. Respiratory and neurologic examinations were normal and RT-PCR SARS-CoV-2 in a nasopharyngeal sample was negative. Antimicrobial empiric treatment was started intravenously with no clinical response. On day 5, the patient developed hypoxemia which needed oxygen therapy flow at 4 L/min and was transferred to an intensive care unit. Given concern for prolonged SARS-CoV-2 infection, induced BAL was performed and COVID-19 was diagnosed from results of a RT-PCR panel. Treatment began with oral steroids (dexamethasone, 6 mg daily), then intravenous (IV) tocilizumab (one injection of 8 mg) and remdesivir IV (200 mg was administered, followed by 100 mg daily for a total of five days) with no response and the persistence of hypoxemia. A high-titer of Omicron CCP (four units) was administered with total regression of symptoms.

### 3.2. Case 2

A 65-year-old female with a past history of multiple sclerosis treated with ocrelizumab sought care for a persistent non-productive cough and dyspnea four months after a SARS-CoV-2 infection. Pulmonary auscultation found unilateral crackling sounds, confirmed on imaging findings. Although RT-SARS-CoV-2 PCR on nasopharyngeal swab was negative, positive PCR results were obtained from the BAL sample, with persistent presence of the SARS-CoV-2 in the lower respiratory tract. She received only IV high-titer Omicron CCP (>8000 IU). A significant clinical improvement was observed, followed by complete resolution of the cough and dyspnea without relapse after 6 months of follow-up.

### 3.3. Case 3

A 48-year-old woman with a history of multiple sclerosis treated with ocrelizumab sought care for a fever which had persisted for 3 months and respiratory symptoms such as a dry cough and dyspnea. Supportive treatment was initiated (analgesics, antitussives) with empirical antimicrobial drugs (amoxicillin/clavulanate, 3 g by IV infusion per day) with no recovery. On admission, she was febrile and had unilateral crackling sounds on pulmonary auscultation. Laboratory findings revealed a high C-reactive protein (CRP) level of 146 mg/L. Nasopharyngeal PCR and serological assay for SARS-CoV-2 were negative. However, we performed a PCR respiratory panel on BAL which detected SARS-CoV-2 RNA. Administrations of four units of high-titer CCP (>8000 IU) were carried out, with a total symptom disappearance and CRP control decrease to 13 mg/L, with no evidence of relapse afterwards.

### 3.4. Case 4

A 61-year-old male, receiving ocrelizumab for multiple sclerosis for 6 months, presented to the emergency room with an approximate 2 months history of daily fever and cough. He received two empiric courses of antibiotics (spiramycine once and amoxicillin-clavulanate twice) for presumed community-acquired pneumonia. A nasopharyngeal swab for SARS-CoV-2 was negative. COVID-19 was diagnosed from the results of RT-PCR on BAL, and CT thoracic imaging (Figure 2). After CCP, oral nirmatrelvir/ritonavir for five days and IV remdesevir (200 mg on the first day, then 100 mg/day for two days) administration outcome was favorable with rapid improvement of general condition and respiratory symptoms.

## 4. Discussion

This case series suggested (i) the use of lower respiratory samples such as bronchoalveolar lavage (BAL) is required in patients with anti-CD20 agents, in case of clinical examination and imaging finding suggesting SARS-CoV-2 infection and (ii) using CCP is a good alternative treatment for patients receiving anti-CD20 agents (such as ocrelizumab and rituxumab) who experience prolonged SARS-CoV-2 infection.

In our patients, repeated nasopharyngeal swabs were negative for SARS-CoV2 RT-PCR, but these negative results on nasopharyngeal swabs are clearly insufficient to rule out COVID-19 diagnosis [2]. We strongly recommend the use of lower respiratory tract specimen testing for the direct detection of SARS-CoV-2 infection in immunosuppressed patients, in case of clinical and/or radiological suspicion [11]. Morandi et al. evaluated the additional value of lower respiratory tract sampling in the diagnosis of COVID-19 in patients with clinically suspected SARS-CoV-2 infection with available chest CT scan and at least two negative nasopharyngeal swab RT-PCR tests for SARS-CoV-2 [12]. A correlation was found between SARS-CoV2 detection on the lower respiratory tract and the presence of a cough as well as with typical CT features. The Fab domains of anti-CD20 mAbs, such as ocrelizumab and rituxumab, target CD20+ B lymphocytes, causing selective depletion of circulating B cells through natural killer cell-mediated, antibody-dependent cellular cytotoxicity, complement-dependent cytotoxicity, and antibody-triggered apoptosis. In all our patients who received B-cell depletion therapy, prolonged SARS-CoV-2 viral shedding was reported with a mean of 48 days, which was consistent with medical literature data [1]. Recently, remdesivir and nirmatrelvir/ritonavir combined with sotrovimab were suggested as treatment in several cases of COVID-19 patients with prolonged clinical symptoms and viral shedding [13,14,15], but also in pre- and post-exposure prophylaxis of COVID-19 [16]. The efficacy of CCP to treat long-standing COVID-19 in patients with B-Cell depletion was discussed [4,17]. In our paper, patient 2 and 3 were treated exclusively with high-titer post-vaccine CCP with a complete recovery. Scott et al. suggested that spike-protein proteolytic antibodies in CCP contribute to SARS-CoV-2 neutralization [18]. Cognasse et al. also demonstrated that CCP exhibited moderately increased inflammatory markers on endothelial cells, neutralizing auto-Abs to type I IFNs compared to the control plasma with no discernible differences in ex vivo bioactivity [3]. CCP therapy with robust Fc-effector antiviral functions can serve as secondary defense when neutralization is compromised [19]. Given the increasingly recognized role of T-cell responses in protection against severe COVID-19, the circulation of SARS-CoV-2 variants, and the potential implementation of novel vaccines, it becomes imperative to continuously monitor T-cell responses [20,21].

Currently, the use of CCP is permitted in France as an off-label indication, and it implies authorization by ‘The National Reference Multidisciplinary Team’. CCP therapy seems to be a promising approach through the transfer of neutralizing antibodies specific to SARS-CoV-2 in patients with B-cell immunosuppression and prolonged COVID-19. Further studies are required to clarify therapeutic management strategies for immunocompromised patients receiving B-cell depletion therapy who experience prolonged COVID-19.

## Figures and Tables

**Table 1:** Demographic data, clinical characteristics, laboratory findings, and prescribed treatments in patients with B-cell depletion and prolonged SARS-CoV-2 infection, Nord Franche-Comté Hospital, 2022–2023.

		Patient 1	Patient 2	Patient 3	Patient 4
***Patients characteristics***					
Sex		F	F	F	M
Age, y		43	65	48	61
Underlying comorbidities		Rheumatoid arthritis	Multiple sclerosis	Multiple sclerosis, diabetes mellitus	Multiple sclerosis
Past history of anti-CD20 mAbs (y)		-	Rituximab (2017–2019)	Rituximab (2017–2019)	Rituximab (2018–2019)
Current anti-CD20 mAbs (y)		Rituximab (since 2016)	Ocrelizumab (since 2019)	Ocrelizumab (since 2019)	Ocrelizumab (since 2019)
Other treatments		Leflunomid, CTC		Sitagliptin	
***Clinical features***					
Clinical presentation	Onset date	15 March 2023	28 September 2022	25 January 2022	28 March 2023
	Symptoms	Fever, sweating, weight loss, dyspnea	Fever with ILI, cough, dyspnea	Fever, asthenia, cough, dyspnea, retrosternal pain	Fever, cough, sweating, dyspnea
Sounds heard on pulmonary auscultation		Without abnormalities	Unilateral crackling (left)	Unilateral crackling (left)	Bilateral crackling
Oxygen support		4 L/min	0 L/min	0 L/min	0 L/min
***Laboratory findings and microbiological findings***					
NP SARS-CoV-2 RT-PCR	Days from onset (date)	56 (10 May 2023)	58 (26 November 2023)	33 (27 February 2023)	46 (13 May 2023)
	Results	Negative	Negative	Negative	Negative
Serology SARS-CoV-2		Negative	Negative	Negative	Negative
White blood cells count (G/L)		4.70	5.15	7.67	11.29
C-reactive protein (mg/L)		57.53	13.44	146.79	50.07
Peripheral Blood cultures		Negative	NA	Negative	Negative
BAL	Days from onset (date)	69 (23 May 2023)	76 (13 December 2023)	40 (6 March 2023)	50 (17 May 2023)
	Culture	Negative	Negative	Negative	Negative
	SARS-CoV-2 RT-PCR Results * (CT if available)	Positive (NA)	Positive (E 33.1-RdRP 35.2-N2 37.0)	Positive (NA)	Positive (QS5 27.8- ORF1ab 27.9-S 27.8)
***Imaging findings***					
Chest X-ray		Bronchial thickening	ND	Interstitial lung, left base condensation	Interstitial lung opacities
Pulmonary CT scan		Bilateral interstitial lung disease, bilateral GGO	Bilateral GGO	Bilateral GGO with a basal distribution	Ground glass and consolidation in middle and lower lobes
***Treatments***					
Antimicrobial drugs		3GC/Piperacillin-tazobactam/Levofloxacine/TMP-SMX	NA	3GC/Amoxicillin clavulanate/Piperacillin-tazobactam/Spiramycine	3GC/Amoxicillin clavulanate/Piperacillin-tazobactam/Spiramycine
Specific treatments (drugs)		Remdesivir/IL6-receptor antagonists (TCZ)/ CTC (DXM)/CCP **	CCP **	CPP **	Remsedevir/Nirmatrelvir-ritonavir/CPP **
***Outcomes***					
Clinical	Recovery	Yes 85 (8 June 2023)	Yes 88 (25 December 2022)	Yes 52 (18 March 2023)	Yes 67 (3 June 2023)
	Resolution of symptoms from onset, in days (date)				
	Follow-up from recovery, in days (last date)	24 (1 July 2023)	186 (30 June 2023)	49 (5 July 2023)	32 (5 July 2023)
Microbiological NP SARS-CoV-2 RT-PCR 7 days after CPP administration ***		Negative	Negative	Negative	Negative

Abbreviations (alphabetic order): 3GC: third-generation cephalosporin; BAL: Broncho alveolar lavage; CCP: COVID-19 convalescent plasma; CT: cycle threshold, CTC: corticosteroids therapy; CT scan: computed tomography scan; DXM: dexamethasone; E: envelope gene; F: female; GGO: ground glass opacities; G/L: giga per liter; L/min: liter per minute; ILI: influenza-like illness; N: nucleocapsid gene; NA: not applicable; NP: Nasopharyngeal; ND: not done; M: male; mAbs: monoclonal antibodies (mAbs); mg/L: milligram per liter; ORF1ab: specific Open Reading Frame; RdRP: ARN polymerase gene; RT-PCR: reverse transcription polymerase chain reaction; S: protein S gene; TCZ: tocilizumab; TMP-SMX: Trimethoprim-sulfamethoxazole; y: years. * We performed in all our patients DiagCORE ^®^, Hong Kong, China, Respiratory Panel 2—SAT Dx., which detects viral and bacterial pathogens including human mastadenovirus A-G (formerly adenovirus), primate bocaparvovirus 1 + 2 (formerly bocavirus), human (hMPV), rhinovirus/enterovirus, influenza A virus (as no subtype, subtype H1, H1N1/2009, or H3), influenza B virus, human respirovirus 1 or 3, human orthorubulavirus 2 or 4 (formerly human parainfluenza virus type 1–4), human orthopneumovirus, *Mycoplasma pneumoniae*, *Legionella pneumophilia*, *Bordetella pertussis* and *Chlamydia pneumoniae*, coronavirus (differentiating HKU1, NL63, OC43, or 229E), human metapneumovirus A/B, and SARS-CoV-2. Amplification curves and cycle threshold (Ct) values were not mentioned in patient 1 and 3. ** We administered four units of high-titer post-vaccine Omicron COVID-19 convalescent plasma in all patients (two units on day 1 and two units on day 2). *** BAL SARS-CoV-2 RT-PCR follow-up after convalescent plasma administration were not performed in any patients regarding the complete recovery.

## Data Availability

Data available on request due to privacy restrictions. The data presented in this case study are available on request from the corresponding author.

## References

[1] Gibson E.G., Pender M., Angerbauer M., Cook C., Jones B., Spivak A.M., Spivak E.S., Swaminathan S. (2021). Prolonged SARS-CoV-2 Illness in a Patient Receiving Ocrelizumab for Multiple Sclerosis. Open Forum Infect. Dis..

[2] Winichakoon P., Chaiwarith R., Liwsrisakun C., Salee P., Goonna A., Limsukon A., Kaewpoowat Q. (2020). Negative Nasopharyngeal and Oropharyngeal Swabs Do Not Rule Out COVID-19. J. Clin. Microbiol..

[3] Cognasse F., Hamzeh-Cognasse H., Rosa M., Corseaux D., Bonneaudeau B., Pierre C., Huet J., Arthaud C.A., Eyraud M.A., Prier A. (2023). Inflammatory markers and auto-Abs to type I IFNs in COVID-19 convalescent plasma cohort study. EBioMedicine.

[4] Tomisti L., Angelotti F., Lenzi M., Amadori F., Sarteschi G., Porcu A., Capria A.-L., Bertacca G., Lombardi S., Bianchini G. (2023). Efficacy of Convalescent Plasma to Treat Long-Standing COVID-19 in Patients with B-Cell Depletion. Life.

[5] D’Abramo A., Vita S., Maffongelli G., Mariano A., Agrati C., Castilletti C., Goletti D., Ippolito G., Nicastri E. (2021). Prolonged and severe SARS-CoV-2 infection in patients under B-cell-depleting drug successfully treated: A tailored approach. Int. J. Infect. Dis..

[6] Tortorella C., Aiello A., Gasperini C., Agrati C., Castilletti C., Ruggieri S., Meschi S., Matusali G., Colavita F., Farroni C. (2022). Humoral- and T-Cell-Specific Immune Responses to SARS-CoV-2 mRNA Vaccination in Patients with MS Using Different Disease-Modifying Therapies. Neurology.

[7] Aiello A., Coppola A., Ruggieri S., Farroni C., Altera A.M.G., Salmi A., Vanini V., Cuzzi G., Petrone L., Meschi S. (2023). Longitudinal characterisation of B and T-cell immune responses after the booster dose of COVID-19 mRNA-vaccine in people with multiple sclerosis using different dis-ease-modifying therapies. J. Neurol. Neurosurg. Psychiatry.

[8] Dynamic Evolution of Humoral and T-Cell Specific Immune Response to COVID-19 mRNA Vaccine in Patients with Multiple Sclerosis Followed until the Booster Dose—PubMed. https://pubmed.ncbi.nlm.nih.gov/37239872/.

[9] Petrone L., Tortorella C., Aiello A., Farroni C., Ruggieri S., Castilletti C., Meschi S., Cuzzi G., Vanini V., Palmieri F. (2022). Humoral and Cellular Response to Spike of Delta SARS-CoV-2 Variant in Vaccinated Patients with Multiple Sclerosis. Front. Neurol..

[10] Aiello A., Coppola A., Vanini V., Petrone L., Cuzzi G., Salmi A., Altera A.M.G., Tortorella C., Gualano G., Gasperini C. (2022). Accuracy of QuantiFERON SARS-CoV-2 research use only assay and characterization of the CD4+ and CD8+ T cell-SARS-CoV-2 response: Comparison with a homemade interferon-γ release assay. Int. J. Infect. Dis..

[11] Puyskens A., Michel J., Stoliaroff-Pepin A., Bayram F., Sesver A., Wichmann O., Harder T., Schaade L., Nitsche A., Peine C. (2023). Direct comparison of clinical diagnostic sensitivity of saliva from buccal swabs versus combined oro-/nasopharyngeal swabs in the detection of SARS-CoV-2 B.1.1.529 Omicron. J. Clin. Virol. Off. Publ. Pan Am. Soc. Clin. Virol..

[12] Morandi L., Torsani F., Forini G., Tamburrini M., Carnevale A., Pecorelli A., Giganti M., Piattella M., Guzzinati I., Papi A. (2022). The Additional Value of Lower Respiratory Tract Sampling in the Diagnosis of COVID-19: A Real-Life Observational Study. Diagnostics.

[13] Nakamura K., Sugiyama M., Ishizuka H., Sasajima T., Minakawa Y., Sato H., Miyazawa M., Kitakawa K., Fujita S., Saito N. (2023). Prolonged infective SARS-CoV-2 omicron variant shedding in a patient with diffuse large B cell lymphoma successfully cleared after three courses of remdesivir. J. Infect. Chemother. Off. J. Jpn. Soc. Chemother..

[14] Lanzafame M., Gottardi M., Guella L., Collini L., Costa G., Guella A., Vento S. (2023). Successful treatment of persistent SARS-CoV-2 infection with nirmatrelvir/ritonavir plus sotrovimab in four immunocompromised patients. J. Chemother..

[15] Baldi F., Dentone C., Mikulska M., Fenoglio D., Mirabella M., Magnè F., Portunato F., Altosole T., Sepulcri C., Giacobbe D.R. (2022). Case report: Sotrovimab, remdesivir and nirmatrelvir/ritonavir combination as salvage treatment option in two immunocompromised patients hospitalized for COVID-19. Front. Med..

[16] Vita S., Rosati S., Ascoli Bartoli T., Beccacece A., D’Abramo A., Mariano A., Scorzolini L., Goletti D., Nicastri E. (2022). Monoclonal Antibodies for Pre- and Postex-posure Prophylaxis of COVID-19: Review of the Literature. Pathogens.

[17] Hueso T., Godron A.-S., Lanoy E., Pacanowski J., Levi L.I., Gras E., Surgers L., Guemriche A., Meynard J.-L., Pirenne F. (2022). Convalescent plasma improves overall survival in patients with B-cell lymphoid malignancy and COVID-19: A longitudinal cohort and propensity score analysis. Leukemia.

[18] McConnell S.A., Sachithanandham J., Mudrak N.J., Zhu X., Farhang P.A., Cordero R.J.B., Wear M.P., Shapiro J.R., Park H.-S., Klein S.L. (2023). Spike-protein proteolytic antibodies in COVID-19 convalescent plasma contribute to SARS-CoV-2 neutralization. Cell Chem. Biol..

[19] Ullah I., Beaudoin-Bussières G., Symmes K., Cloutier M., Ducas E., Tauzin A., Laumaea A., Grunst M.W., Dionne K., Richard J. (2023). The Fc-effector function of COVID-19 con-valescent plasma contributes to SARS-CoV-2 treatment efficacy in mice. Cell Rep. Med..

[20] Goletti D., Petrone L., Manissero D., Bertoletti A., Rao S., Ndunda N., Sette A., Nikolayevskyy V. (2021). The potential clinical utility of measuring severe acute respiratory syndrome coronavirus 2-specific T-cell responses. Clin. Microbiol. Infect..

[21] Petrone L., Sette A., de Vries R.D., Goletti D. (2023). The Importance of Measuring SARS-CoV-2-Specific T-Cell Responses in an Ongoing Pandemic. Pathogens.

